# Scavenging effect of pasipay (*passiflora incarnate* L.) on singlet oxygen generation and fatty acid photooxygenation

**DOI:** 10.1002/fsn3.731

**Published:** 2018-07-20

**Authors:** Mahdi Hajimohammadi, Parisa Nosrati

**Affiliations:** ^1^ Faculty of Chemistry Kharazmi University Tehran Iran

**Keywords:** antioxidant, fatty acid, *Pasipay incarnate* L, reactive oxygen species, singlet oxygen

## Abstract

Anthracene as a chemical probe is usually used to trap the singlet oxygen and then detection and quantification can be based on absorbance. In this study, oxidation of anthracene declared that rate of singlet oxygen quenching in the presence of pasipay (*passiflora incarnate* L.) as a natural antioxidant, 1,4 Diazabicyclo [2.2.2] octane (DABCO) as a well‐known singlet oxygen scavenger and highly effective synthetic antioxidants in food industry such as Butylated hydroxytoluene (BHT), Butylated hydroxyanisole (BHA), tert‐Butylhydroquinone (TBHQ) decreased in the order of DABCO >pasipay > TBHQ > BHT > BHA. On the other hand, lipid photooxidation is the undesirable chemical process in which singlet oxygen result in the peroxidation of fatty acids. The results of this study also showed that oleic acid oxidation with singlet oxygen in the presence of pasipay (contains 0.4576 mg flavonoid compounds) diminished about 11% which shows pasipay has an effective role to inhibit lipid peroxidation.

## 1 INTRODUCTION

Oxygen molecule in its ground state has two unpaired electrons and when oxygen molecule has excess energy, these unpaired electrons in the external orbital can be pair and generate singlet oxygen (Min & Boff, 2002). One of the physical methods for producing singlet oxygen is photosensitization reaction. Great photosensitizers have received attention, due in part to their direct relevance to many biological systems. Electrophilic tendency of singlet oxygen causes lipids, amino acids, nucleic acids, and electron rich molecular can be its target (Korytowski, Schmitt, & Girotti, 2010). Singlet oxygen can be easily produced in food systems under light illumination, especially in the presence of photosensitizers such as riboflavin and chlorophylls (Greer, 2006). Lipids also can be a target of singlet oxygen due to the electrophilic inherent, this reactive species attacked to unsaturated fatty acid and produce lipid hydroperoxides as primary products (Girotti, 1998). On the other hand UV radiation causes DNA damage and protein oxidation and induces the synthesis of matrix metalloproteinases (Kligman, 1989). The use of antioxidants to protect human skin from the harmful effects of UV radiation is a topic that has attracted growing interest in recent years within the world of photoprotection research (Martinez et al., 2017). DABCO recognized as a very efficient quencher of singlet oxygen in the organic media (Lengfelder, Cadenas, & Sies, 1983) and synthetic antioxidants such as TBHQ, BHA, and BHT have been found to have a strong singlet oxygen quenching ability (Lee & Jung, 2010). Recent research has focused on isolation and characterization of effective natural antioxidants (Fang et al., 2017; Nimse & Pal, 2015). Natural antioxidants act (a) as reducing agents, (b) as free radical scavengers, and (c) as quenchers of the formation of singlet oxygen. They can be used in the food industry and there is evidence that they may exert their antioxidant effects within the human body (Hamid, Aiyelaagbe, Usman, Ameen, & Lawal, 2010; Koski et al., 2002). People receive antioxidant supplements directly from fresh fruits and vegetables. The World Health Organization estimated that <80% of the earth's inhabitants rely on traditional medicine for their primary health care needs and most of this therapy involves the use of plant extracts or their active phenolic components (Bruneton, 1995) which have an efficient antioxidant capacity. Pasipay is the largest and most important genus of the family Pasipayceae, comprising about 500 species, distributed mostly in warm temperate and tropical regions (Dhawan, Dhawan, & Sharma, 2004) Previous studies have described the presence of flavonoids as the major constituents of pasipay (Dhawan, Dhawan, & Sharma, 2004). There are few studies on the efficacy of natural antioxidant as O_2_ (^1^Δg) quenchers and their roles in the prevention of lipid oxidation (Niki, 2015; Terao, Minami, & Bando, 2010) because scavenging of DPPH free radical is the basis of a common antioxidant assay and most often an overall antioxidant effect was measured. However, singlet oxygen has not radical nature. (Ruiz‐González et al., 2017) This project was designed to characterize the antioxidant potential of *passiflora incarnate* L. as a natural antioxidant in compare with well‐known singlet oxygen scavenger such as DABCO and the highly effective antioxidants such as BHA, BHT, and TBHQ.

## 2 MATERIAL AND METHODS

### 2.1 Material

Hydroalcoholic extract of *passiflora incarnate* L. was obtained from Iran darouk Co. Anthracene, oleic acid, acetonitrile, methylene blue (MB), DABCO, BHT, BHA, TBHQ were purchased from Fluka and Merck and used without further purification. Tetraphenyl porphyrin (H_2_TPP), ZnTPP, and MgTPP were synthesized according to the literatures (Lindsey & Wagner, 1989).

### 2.2 Method

#### 2.2.1 Sample preparation for anthracene oxidation with singlet oxygen

In a typical experiment, 15 ml acetonitrile, 0.002 mmol antioxidant (DABCO, BHT, BHA, TBHQ, and pasipay (contains 0.5731 mg flavonoid) separately, were added to anthracene (4 × 10^−4^ M) and MB (1 × 10^−4^ M). Continuous irradiation of samples was carried out using solar simulator light (288 power LED lamps, 1 W, 2.3 V (59660 LUX)) for 5 min at room temperature under 1 atm of bubbling of air in the solution at room temperature. Determination of products was recorded on a Shimadzu 2100 spectrophotometer at 375 nm.

#### 2.2.2 Sample preparation for photooxygenation of fatty acid with singlet oxygen

0.0016 mmol antioxidant 7 ml acetonitrile, (DABCO, BHT, BHA, TBHQ and pasipay (contains 0.4576 mg flavonoid) separately, were added to oleic acid (4.6 × 10^−3^ M) and H_2_TPP (1 × 10^−3^ M). Continuous irradiation of samples was carried out using solar simulator light (288 power LED lamps, 1 W, 2.3 V (59660 LUX)) for 120 min at room temperature under 1 atm of bubbling of air in the solution. Determination of products and were analyzed on a Bruker AMX 300 MHz spectrometer using TMS as internal standard and iodometric titration method. Peroxide value (PV) (meq O_2_/kg) of the samples was determined according to the literature (Barthel & Grosch, 1974).

### 2.3 Statistical analysis

In all analyses, three replicates were applied and analysis of the results was achieved using SAS software, version 3.9 and then average the results were compared using Duncan test. Also, with Excel software diagrams were drawn.

## 3 RESULTS AND DISCUSSION

### 3.1 Evidences for singlet oxygen generation and biocidal activity of pasipay in the photooxidation of oleic acid

**Figure 1:**
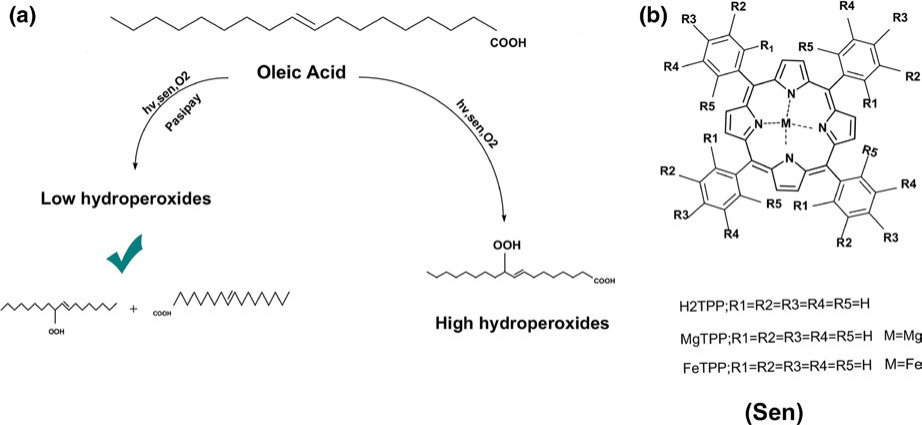
Oleic acid photooxygenation in the presence and absence of Pasipay (a). Structure of different applied photosensitizers (b)

In this work, the oxidative alterations of oleic acid as a result of oxidation with singlet oxygen were analyzed in the presence and absence of pasipay as a source of natural phenolic compounds. Our target was fatty acid oxidation with singlet oxygen as a noble species which has worked few studies on it (Terao, Minami, & Bando, 2010). Photooxygenation of oleic acid with H_2_TPP as a photosensitizers was investigated as a typical standard sample to evaluate singlet oxygen production (Figure 1).

**Figure 2:**
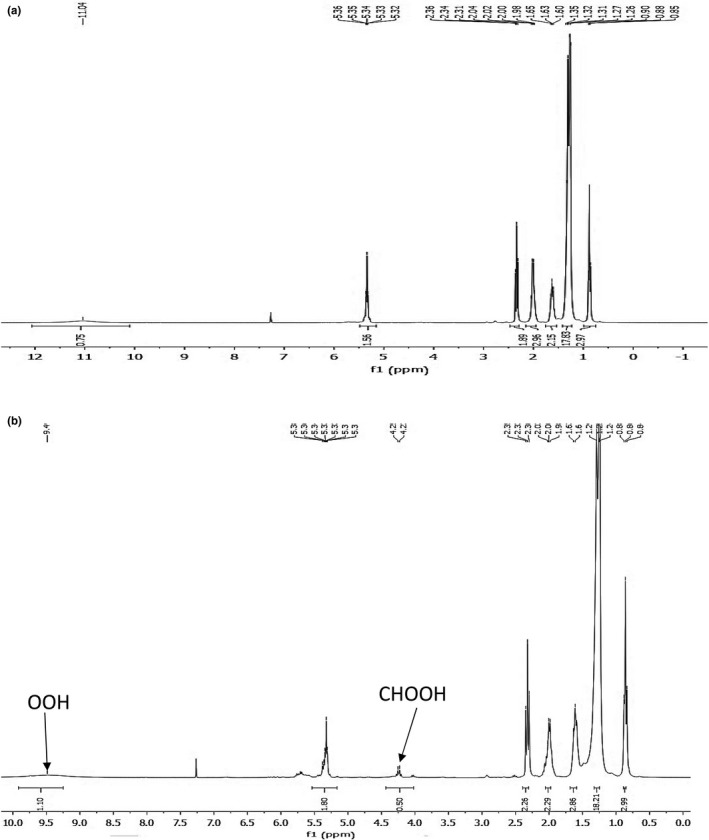
^1^H NMR spectra of oleic acid (3.1 × 10^−3^ mol) after photooxidation in the absence (a) and in the presence (b) of H_2_ TPP (0.001M)

It is important to note that ^1^H NMR spectroscopy and iodometric method revealed oxidation of oleic acid to peroxide product stopped in the absence of photosensitizers (Figure 2 and Table 1 entry 1) or when the irradiation was interrupted (Table 1 entry 2). Accordingly, the presence of a H_2_TPP, light and O_2_ are essential for the conversion oleic acid to corresponding products (Table 1 entry 3).

**Table 1:** PV of oleic acid oxidation with singlet oxygen in different condition^a^

Entry	Condition	PV
1	Oleic acid + CH_3_CN + light + air	Trace
2	Oleic acid + CH_3_CN + air + H_2_TPP	Trace
3	Oleic acid + CH_3_CN + air + H_2_TPP + ight	101.67
4	Oleic acid + CH_3_CN + light + air + ZnTPP	79.33
5	Oleic acid + CH_3_CN + light + air + MgTPP	70.39
6	Oleic acid + CH_3_CN + H_2_TPP + light + DABCO	Trace
7	Oleic acid + H_2_TPP + light + air + DMSO	27.93
8^b^	Oleic acid + H_2_TPP + light + air + Ethanol	89.38
9	Oleic acid + $O_{2}^{-}$	Trace

^a^4.6⨯10^−3^ mol oleic acid, 5 ml solvent, 1 ml (0.001 M) sensitizer, air (1 atm) and 288 power LED lamps, 1 W, 2.3 V (59660 LUX). ^b^ $O_{2}^{-}$ was prepared by dissolving K_2_O in dried DMSO.

**Figure 3:**
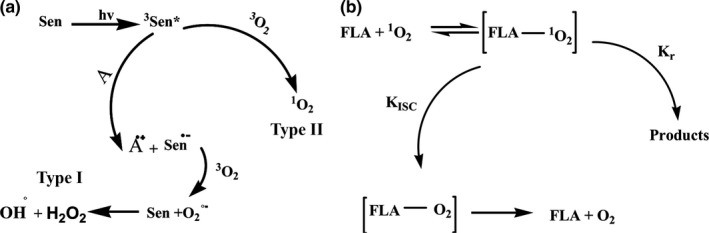
The mechanisms for producing reactive oxygen species in the presence of photosensitizers (a) The mechanism of flavonoids (FLA) barricade of Pasipay against singlet oxygen (b)

According to the literature, there are two major pathways for photooxygenation reactions in the presence of nonmetal photosensitizers, Type I and Type II (Figure 3a) (Laing, 1989).

Singlet oxygen generation (Type II) and its reaction with the substrates is the foremost mechanism that occurs in our circumstances, since conversions of oleic acid obey the order of H_2_TPP > ZnTPPCl > MgTPPCl (Table 1 entry 3, 4, and 5). Paramagnetic metals are claimed to quench singlet oxygen by energy transfer mechanism from oxygen to the low‐lying d orbital electron levels and have very short triplet lifetimes (Table 1, entry 4) (Bonnett & Martınez, 2001) also diamagnetic metals quench singlet oxygen by a charge transfer mechanism (Bonnett & Martınez, 2001) (Table 1, entry 5). In addition, in the presence of DABCO, which is a well‐known singlet oxygen scavenger (Lengfelder et al., 1983) (Table 1, entry 6) photooxidation of oleic acid was inhibited. According to the literature, singlet oxygen lifetime in dimethyl sulfoxide (DMSO) solvent is 19 μs, in acetonitrile (CH_3_CN) solvent is 65 μs, and in ethanol solvent is 38 μs which was corresponded with the results in Table 1 (entry 3,7, and 8) (Bressan & Morvillo, 1989; Chen et al., 2001; Toffoli, Gomes, Junior, & Courrol, 2008). Table 1 entry 3, 7, and 8 indicates that conversion of oleic acid in acetonitrile as solvent is higher than ethanol and DMSO that correlated with singlet oxygen lifetimes in this solvents. For investigation of the type I mechanism, we performed our reaction in the presence superoxide anion radical (O_2_^•–^). In the presence of O_2_^•–^, the rates of oxidation reaction significantly decreased (Table 1 entry 9).

### 3.2 Effect of pasipay on Anthracene photooxygenation

**Figure 4:**
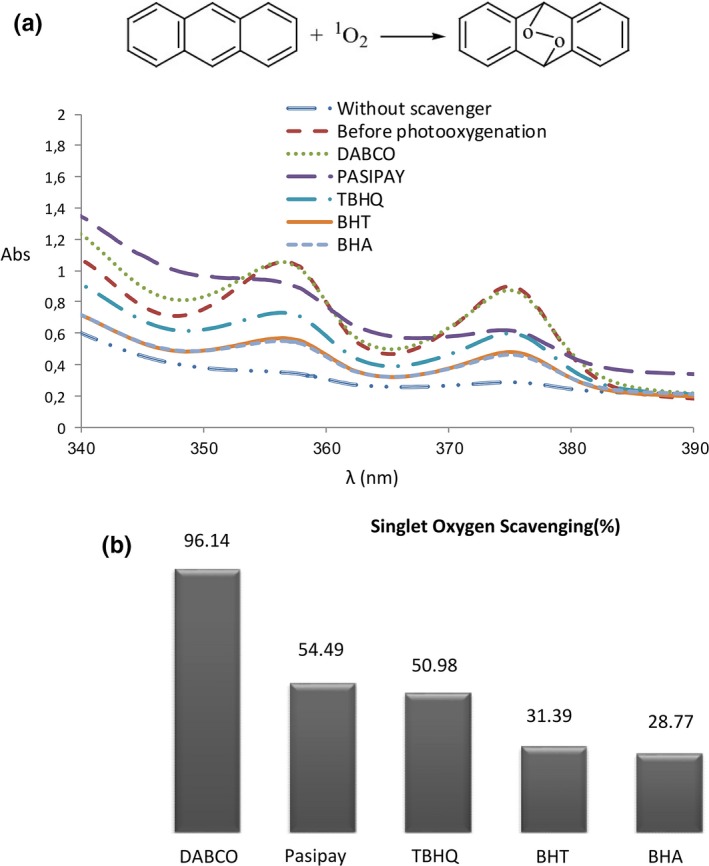
UV–visible spectra of anthracene photooxygenation with singlet oxygen in the presence of different kind of singlet oxygen scavengers (λmax = 375 nm) after 5 min using solar simulator light (288 power LED lamps, 1 W, 2.3 V (59660 LUX)) under 1 atm of bubbling of air in the acetonitryl (a) The scavenging capacity of different kind of singlet oxygen scavengers after 5 min using solar simulator light (288 power LED lamps, 1 W, 2.3 V (59660 LUX)) under 1 atm of bubbling of air in the acetonitrile

Spectrophotometry is a more convenient option for detection of excited oxygen molecules. A chemical probe is usually used to trap the singlet oxygen and then detection and quantification can be based on absorbance. A very characteristic reaction of singlet oxygen is the [4 + 2] cycloaddition to conjugated cyclic dienes and polycyclic aromatic hydrocarbons such as anthracene (Aubry, Pierlot, Rigaudy, & Schmidt, 2003). Anthracene traps reversibly singlet oxygen. Singlet oxygen generation by MB is evidenced by chemical trapping of ^1^O_2_ with anthracene. The UV–Vis spectra of anthracene as the function of time irradiation by using of MB as a photosensitizer are displayed in Figure 4a. A reduction of the emission intensity absorption band of anthracene (λmax = 375 nm) was observed with the increase in irradiation time. This response is a consequence of the anthracene‐9,10‐endoperoxide formation (see Figure 4). During the reaction, the addition of well‐known singlet oxygen scavenger such as DABCO and highly effective synthetic antioxidants in food industry in foods such as BHT, BHA, TBHQ, and pasipay inhibited the oxidation of anthracene in the order of DABCO > pasipay > TBHQ > BHT > BHA (Figure 4a,b). Moreover, the oxidation reaction did not occur under dark conditions. This confirms that the anthracene oxidation occurs by singlet oxygen under visible irradiation. These results show pasipay act as a very effective deterrent on singlet oxygen generation.

### 3.3 Effect of pasipay on fatty acid photooxgenation

**Figure 5:**
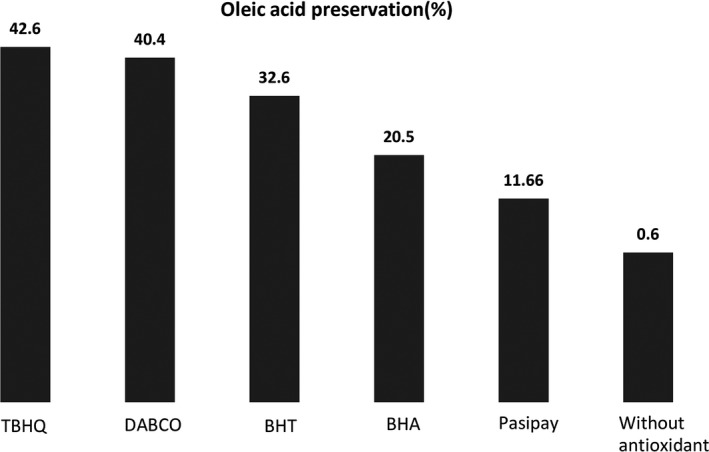
Diagram of oleic acid preservation in the presence of pasipay and well‐known fatty acid antioxidants (BHA, BHT and TBHQ) and chemical singlet oxygen scavenger (DABCO)

The photosensitized production of singlet oxygen has significance in the areas of the photooxidation of organic compounds (Hajimohammadi, Schwarzinger, & Knör, 2012) and food chemistry [1, 2, 4]. Photooxygenation of oleic acid as a one of the targets of singlet oxygen was investigated to evaluate the antioxidant effect of pasipay. Figure 5 shows the conversion of oleic acid to the peroxide products in an oxygenated solution of acetonitrile and H_2_TPP photosensenisitizer under visible light in the presence of pasipay, well‐known singlet oxygen (DABCO) and highly effective antioxidants such as BHT, BHA and TBHQ. After 120 min irradiation, the rate of oleic acid oxidation by ^1^O_2_ as a very reactive ROS reduced 11.6% in the presence of pasipay (contains 0.4576 mg flavonoid compounds) that shows pasipay can be used as an effective additive to fatty acids for the preservation of them. Also, the rate of oleic acid preservation in the presence of these types of antioxidants decreased in the order of TBHQ > DABCO > BHT > BHA > pasipay.

Herbal and natural source of flavonoid and polyphenol compounds have been reported to act as scavengers of various oxidizing species (Dulf, Vodnar, & Socaciu, 2016). On the other hand according to the literature flavonoid compounds trap singlet oxygen and produce FLA‐O_2_ compound (Majer, Neugart, Krumbein, Schreiner, & Hideg, 2014) (Figure 3b). Pasipay because of its flavonoid compounds can use as a highly efficient singlet oxygen scavenger.

## 4 CONCLUSION

Due to the increase of diseases such as cancer, Alzheimer's disease, skin disorders, etc. with ROS especially singlet oxygen and light, finding efficient antioxidant is very important. In this study, antioxidant capacity for pasipay and synthetic polyphenolics against singlet oxygen was comprehensively assessed by anthracene oxidation assay and evaluation of fatty acid oxidation. It was showed pasipay has an effective role in restricting of singlet oxygen generation and limitation of fatty acid photooxidation.

## CONFLICTS OF INTEREST

The authors declare that they have no conflict of interest.

## ETHICAL STATEMENT

This article does not contain any studies with human participants or animals performed by any of the authors.

## References

[fsn3731-bib-0001] Aubry, J. M. , Pierlot, C. , Rigaudy, J. , & Schmidt, R. (2003). Reversible binding of oxygen to aromatic compounds. Accounts of chemical research, 36, 668–675. 10.1021/ar010086g 12974650

[fsn3731-bib-0002] Barthel, G. , & Grosch, W. (1974). Peroxide value determination comparison of some methods. Journal of the American Oil Chemists Society, 51, 540–544. 10.1007/BF02636025

[fsn3731-bib-0003] Bonnett, R. , & Martınez, G. (2001). Photobleaching of sensitisers used in photodynamic therapy. Tetrahedron, 57, 9513–9547. 10.1016/S0040-4020(01)00952-8

[fsn3731-bib-0004] Bressan, M. , & Morvillo, A. (1989). Alkene epoxidation by ruthenium (II) phosphine complexes A kinetic investigation. Inorganic Chemistry, 28, 950–953. 10.1021/ic00304a028

[fsn3731-bib-0005] Bruneton, J. (1995). Pharmacognosy, phytochemistry, medicinal plants. Paris: Lavoisier publishing.

[fsn3731-bib-0006] Chen, Y. , Xu, S. , Li, L. , Zhang, M. , Shen, J. , & Shen, T. (2001). Active oxygen generation and photo‐oxygenation involving temporfin (m‐THPC). Dyes and pigments, 51, 63–69. 10.1016/S0143-7208(01)00071-7

[fsn3731-bib-0007] Dhawan, K. , Dhawan, S. , & Sharma, A. (2004). Passiflora: A review update. Journal of ethnopharmacology, 94, 1–23. 10.1016/j.jep.2004.02.023 15261959

[fsn3731-bib-0008] Dulf, F. V. , Vodnar, D. C. , & Socaciu, C. (2016). Effects of solid‐state fermentation with two filamentous fungi on the total phenolic contents, flavonoids, antioxidant activities and lipid fractions of plum fruit (*Prunus domestica L*.) by‐products. Food chemistry, 209, 27–36. 10.1016/j.foodchem.2016.04.016 27173530

[fsn3731-bib-0009] Fang, T. , Wu, X. , Cao, W. , Jia, G. , Zhao, H. , Chen, X. , … Liu, G. (2017). Effects of dietary fiber on the antioxidant capacity, immune status, and antioxidant‐relative signaling molecular gene expression in rat organs. Royal Science Chemistry Advances, 7(7), 19611–19620. 10.1039/C7RA02464A

[fsn3731-bib-0010] Girotti, A. W. (1998). Lipid hydroperoxide generation, turnover, and effector action in biological systems. Journal of lipid research, 39, 1529–1542. 9717713

[fsn3731-bib-0011] Greer, A. (2006). Christopher Foote's discovery of the role of singlet oxygen [^1^O_2_ (^1^Δg)] in photosensitized oxidation reactions. Accounts of chemical research, 39, 797–804. 10.1021/ar050191g 17115719

[fsn3731-bib-0012] Hajimohammadi, M. , Schwarzinger, C. , & Knör, G. (2012). Controlled multistep oxidation of alcohols and aldehydes to carboxylic acids using air, sunlight and a robust metalloporphyrin sensitizer with a pH‐switchable photoreactivity. Royal Science Chemistry Advances, 2, 3257–3260. 10.1039/C2RA01076C

[fsn3731-bib-0013] Hamid, A. A. , Aiyelaagbe, O. O. , Usman, L. A. , Ameen, O. M. , & Lawal, A. (2010). Antioxidants: its medicinal and pharmacological applications. African Journal of pure and applied Chemistry, 4, 142–151.

[fsn3731-bib-0014] Kligman, L. H. (1989). Prevention and repair of photoaging: Sunscreens and retinoids. Cutis, 43, 458–465. 2656109

[fsn3731-bib-0015] Korytowski, W. , Schmitt, J. C. , & Girotti, A. W. (2010). Surprising Inability of Singlet Oxygen‐generated 6‐Hydroperoxycholesterol to Induce Damaging Free Radical Lipid Peroxidation in Cell Membranes. Photochemistry and photobiology, 86, 747–751. 10.1111/j.1751-1097.2010.00722.x 20408976 PMC2910147

[fsn3731-bib-0016] Koski, A. , Psomiadou, E. , Tsimidou, M. , Hopia, A. , Kefalas, P. , Wähälä, K. , & Heinonen, M. (2002). Oxidative stability and minor constituents of virgin olive oil and cold‐pressed rapeseed oil. European Food Research and Technology, 214, 294–298. 10.1007/s00217-001-0479-5

[fsn3731-bib-0017] Laing, M. (1989). The three forms of molecular oxygen. Journal of Chemical Education, 66, 453 10.1021/ed066p453

[fsn3731-bib-0018] Lee, J. H. , & Jung, M. Y. (2010). Direct spectroscopic observation of singlet oxygen quenching and kinetic studies of physical and chemical singlet oxygen quenching rate constants of synthetic antioxidants (BHA, BHT, and TBHQ) in methanol. Journal of food science, 75, 10.1111/j.1750-3841.2010.01669.x 20722904

[fsn3731-bib-0019] Lengfelder, E. , Cadenas, E. , & Sies, H. (1983). Effect of DABCO (1, 4‐diazabicyclo [2, 2, 2]‐octane) on singlet oxygen monomol (1270 nm) and dimol (634 and 703 nm) emission. FEBS letters, 164, 366–370. 10.1016/0014-5793(83)80318-4

[fsn3731-bib-0020] Lindsey, J. S. , & Wagner, R. W. (1989). Investigation of the synthesis of ortho‐substituted tetraphenylporphyrins. Journal of Organic Chemistry, 54, 828–836. 10.1021/jo00265a021

[fsn3731-bib-0021] Majer, P. , Neugart, S. , Krumbein, A. , Schreiner, M. , & Hideg, É. (2014). Singlet oxygen scavenging by leaf flavonoids contributes to sunlight acclimation in *Tilia platyphyllos* . Environmental and Experimental Botany, 100, 1–9. 10.1016/j.envexpbot.2013.12.001

[fsn3731-bib-0022] Martinez, R. M. , Pinho‐Ribeiro, F. A. , Steffen, V. S. , Caviglione, C. V. , Fattori, V. , Bussmann, A. J. C. , … Baracat, M. M. (2017). Trans‐Chalcone, a Flavonoid Precursor, Inhibits UV‐Induced Skin Inflammation and Oxidative Stress in Mice by Targeting NADPH Oxidase and Cytokine Production. Photochemical & Photobiological Sciences, 16, 1162–1173. 10.1039/c6pp00442c 28594010

[fsn3731-bib-0023] Min, D. B. , & Boff, J. M. (2002). Chemistry and reaction of singlet oxygen in foods. Comprehensive Reviews in Food Science and Food Safety, 1, 58–72. 10.1111/j.1541-4337.2002.tb00007.x 33451241

[fsn3731-bib-0024] Niki, E. (2015). Lipid oxidation in the skin. Free radical research, 49, 827–834. 10.3109/10715762.2014.976213 25312699

[fsn3731-bib-0025] Nimse, S. B. , & Pal, D. (2015). Free radicals, natural antioxidants, and their reaction mechanisms. Royal Science Chemistry Advances, 5, 27986–28006. 10.1039/C4RA13315C

[fsn3731-bib-0026] Ruiz‐González, R. , Bresolí‐Obach, R. , Gulías, Ò. , Agut, M. , Savoie, H. , Boyle, R. W. , … Giuntini, F. (2017). NanoSOSG: A nanostructured fluorescent probe for the detection of intracellular singlet oxygen. Angewandte Chemie International Edition, 56, 2885–2888. 10.1002/anie.201609050 28151569

[fsn3731-bib-0027] Terao, J. , Minami, Y. , & Bando, N. (2010). Singlet molecular oxygen‐quenching activity of carotenoids: Relevance to protection of the skin from photoaging. Journal of Clinical Biochemistry and Nutrition, 48, 57–62. 10.1002/anie.201609050 21297913 PMC3022065

[fsn3731-bib-0028] Toffoli, D. J. , Gomes, L. , Junior, N. D. V. , & Courrol, L. C. (2008). Enhancement on the hypocrellin B singlet oxygen generation quantum yield in the presence of rare earth ions. AIP Conference Proceedings, 992, 1207–1212. 10.1063/1.2926819

